# Identification of m6A suppressor EIF4A3 as a novel cancer prognostic and immunotherapy biomarker through bladder cancer clinical data validation and pan-cancer analysis

**DOI:** 10.1038/s41598-023-43500-4

**Published:** 2023-09-30

**Authors:** Huaqing Yan, Liqi Zhang, Rubing Li

**Affiliations:** 1 Department of Urology, Ningbo Medical Center Lihuili Hospital, Ningbo, 315000 Zhejiang People’s Republic of China; 2 Department of Reproductive Medicine, The First Affiliated Hospital of Ningbo University, Ningbo, 315000 Zhejiang People’s Republic of China

**Keywords:** Cancer, Genomics

## Abstract

EIF4A3 represents a novel m6A suppressor that exerts control over the global m6A mRNA modification level, therefore influencing gene destiny. Despite increasing evidence that highlights a pivotal role of EIF4A3 in tumor progression and immunity, a comprehensive pan-cancer analysis of EIF4A3 has yet to be conducted, in order to ascertain whether EIF4A3 could be a viable biomarker for cancer screening, prediction of prognosis, and to facilitate accurate therapy design in various human malignancies. We analyzed the expression levels of EIF4A3 in bladder cancer compared to para-cancer tissue. Subsequently survival analysis was conducted to ascertain the potential association between EIF4A3 expression and patient prognosis. To further corroborate this evidence, we conducted an extensive data mining process of several publicly available databases, including UCSC Xena database, TCGA, and GTEx. Raw data from the UCSC Xena database was processed using online tools to obtain results that could be subjected to further analysis. Our study unveiled a considerable increase in the expression levels of EIF4A3 in bladder cancer compared to para-cancer tissue. Subsequent validation experiments confirmed that bladder cancer patients exhibiting higher levels of EIF4A3 expression have significantly worse prognostic outcomes. Next, our pan-cancer analysis found that the expression level of EIF4A3 is significantly higher in most cancers. Notably, high expression levels of EIF4A3 were negatively associated with patient prognosis across various cancer types. Furthermore, as a novel m6A suppressor, EIF4A3 was found to be correlated with numerous RNA modification genes in multiple cancer types. Meanwhile, analysis of publicly available databases revealed that EIF4A3 expression was significantly related to immune score and immune cell levels in most cancer types. Interestingly, EIF4A3 was also identified as a superior immunotherapy biomarker when compared to several traditional immunotherapy biomarkers. Lastly, genetic alterations analysis revealed that amplification was the most frequently occurring abnormality in the EIF4A3 gene. EIF4A3 emerges as a promising biomarker with the potential to significantly enhance tumor screening, prognostic evaluation, and the design of individualized treatment strategies across a diverse array of malignancies.

## Introduction

Globally, cancer continues to be the leading cause of morbidity and mortality, resulting in a significant burden on public health and economics. Approximately 5365 new cases of cancer are expected to be diagnosed each day in the United States, with a total of 1,958,310 new cases estimated for 2023^1^. Bladder cancer is one of the most common malignant tumors in the urinary system and remains the second most frequent cause of urinary cancer death. While nonmuscle-invasive bladder cancer (NMIBC) account for almost 70% of new bladder cancer diagnoses, approximately 15–20% patients progress to muscle-invasive bladder cancer with a poor prognosis^2^. Within a year, 40–80% of newly diagnosed NMIBC patients undergoing treatment will encounter recurrence^3^. Eventually, around half of patients experience disease in distant areas due to the spread of micrometastases^4^. As a result, systemic treatment is crucial in combination with local therapy to decrease the likelihood of recurrence^5^. Apart from platinum-based chemotherapy as the first-line option, novel therapies including immunotherapy and targeting therapy are providing new hope for advanced bladder cancer patients. So it is important to expand on recent accomplishments by advancing new treatments to earlier stages of disease, refining the use of approved therapies in combination, enhancing patient selection, and discovering new therapeutic targets.

The tumor immune microenvironment (TIME) and tumor cells have a close relationship and influence each other. As the tumor progresses, tumor cells shape an immunosuppressive TIME, which allows them to escape immune surveillance. Meanwhile, an immunosuppressive TIME promotes tumor growth through various mechanisms, such as depleting tumor-infiltrating T cells, the inhibitory role of immune checkpoint genes like VISTA, TIM-3, and LAG-3, and inhibitory immune cells like Tregs, TAMs, and MDSCs^6^. In order to develop more effective cancer treatment strategies, it is essential to study both tumor cells and the tumor immune microenvironment.

Eukaryotic translation initiation factor 4A3 (EIF4A3), as a protein coding gene, is located on chromosome 17q25.3 with 12 exons. The protein encoded by this gene is a member of the DEAD box protein family, which are putative RNA helicases characterized by the conserved motif Asp-Glu-Ala-Asp (DEAD). Meanwhile as the main component of exon junction complex (EJC), EIF4A3 is essential for controlling gene expression through various mechanisms including alternative splicing, translation, mRNA localization, and nonsense-mediated decay. To be of interest, EJCs act as suppressors of m6A specificity, preventing methylation in unmethylated regions of transcripts. This regulation is global and protects exon junction-proximal RNA within coding sequences from methylation, ultimately regulating mRNA stability through m6A suppression^7^. Thus a strong negative correlation between global mRNA m6A modification level and EIF4A3 expression level was identified in 25 human tissues^7^. Finally, loss of EIF4A3 accounts for m6A hypermethylation and modulate gene fate^7,8^. Based on the critical role of EIF4A3 in epigenetics, EIF4A3 was found to regulate cancer progression in different types of human cancer. In ovarian cancer, EIF4A3 was determined to promote the cell viability by cell counting kit-8 and colony formation assays through stabilizing PDK4 mRNA^9^. In Glioblastoma, EIF4A3 can enhance the stability of LINC00680 and TTNAS1, facilitate the cyclization of circMMP9, and upregulate its expression, thereby promoting tumor proliferation, migration, and invasion^10,11^. Similarly, in bladder cancer EIF4A3 was significantly upregulated and related to poor prognosis; knockdown of EIF4A3 significantly inhibited bladder cancer cell proliferation and promoted cell apoptosis^12^.

In addition to its oncogenic function in cancer, EIF4A3 also impacts the immune microenvironment. Recently Hu et al. reported that EIF4A3 promoted the biogenesis of circCCAR1 which was taken in by CD8+T cells and caused dysfunction of CD8+T cells by stabilizing the PD-1 protein, resulting resistance to anti-PD1 immunotherapy^9^. In this study, we validated that EIF4A3 is upregulated in bladder cancer and higher expression of EIF4A3 was significantly associated with a poorer outcome in bladder cancer patients. Although EIF4A3 has been shown to be upregulated in bladder cancer and associated with poor outcomes in patients, its prognostic value and role in immunity across various human cancers remains unclear. Therefore, a systematic pan-cancer analysis of EIF4A3 is urgently needed to determine its potential as a biomarker for cancer screening, prognosis prediction, and therapy design. Our team aims to investigate the association between EIF4A3 and tumor progression and immune microenvironments to determine its potential as a biomarker for human cancers, particularly bladder cancer.

## Materials and methods

### Clinical sample acquisition and immunohistochemical stainning

We purchased 62 bladder cancer tissues and 12 adjacent para-cancer tissues with clinical data from Xinchao Biotech, Shanghai, China. Rabbit polyclonal EIF4A3 antibody was bought from Proteintech, China (Catalog Number 17504-1-AP). The immunohistochemical stainning process was conducted as following: Insert the tissue slide into the oven and set the temperature to 63 °C for 1 h. After the slides are baked, remove them from the oven and place them into an automated staining machine (Dako North America, Autostainer Link 48) for dewaxing. Utilize the DAKO automated immunohistochemistry pre-treatment system (Dako North America, PT Link) for instrument repair. After repair, remove the slides and rinse them three times with PBST buffer, each time for 1 min. Retrieve the primary antibody from the refrigerator and place it into a centrifuge at a speed of 7200 rpm for 30 s. Remove the primary antibody and dilute it with antibody dilution solution in a 1:500 ratio. Add the primary antibody to the slides, incubate at room temperature, and store overnight in a refrigerator at 4 °C. Rinse the slides three times with PBST buffer, each time for 1 min. Retrieve the DAB reagent kit (Dako, Catalog Number K8002) from the refrigerator and prepare it by mixing 1 ml of DAB dilution solution with 1 drop of DAB chromogen. Add the diluted DAB solution to the slides and observe the staining intensity. Allow the staining process for 10 min, then rinse with tap water for 5 min. Add hematoxylin (Sigma aldrich, Catalog Number SLBT4555) to the slides for 1 min, then immerse them in 0.25% hydrochloric acid alcohol (400 ml of 70% alcohol + 1 ml of concentrated hydrochloric acid) for at least 2 s. Rinse with tap water for more than 2 min, air dry at room temperature, and seal the slides. The DAB staining was analyzed using Aperio XT Scanner (LEICA) and ImageScope 12.4 software. To stratify the study cohort into EIF4A3 high and low expression groups, we implemented a standardized approach for processing the original data, as follows:

1. The staining intensity index was categorized into four levels: 0 points (negative), 1 point (1 +), 2 points (2 +), and 3 points (3 +).
2. The positive rate of staining was classified into five levels: 0 points (negative), 1 point (1–25%), 2 points (26–50%), 3 points (51–75%), and 4 points (76–100%).
3. The total score was calculated by multiplying the scores of staining intensity and positive rate. Participants with a total score less than 8 were assigned to the low expression group, while those with a score greater than or equal to 8 were assigned to the high expression group.

In line with the aforementioned approach, the expression levels of CD8 and PDL1 were stratified based on the rates of positive staining. A CD8-positive staining rate of >  = 3% was assigned to the high-expression group, and likewise, a PDL1-positive staining rate of >  = 5% was designated as the high-expression group. The assignment of groups was determined upon the distribution of the data.

### Statistical analysis

Prism 7 (GraphPad Software, Inc., La Jolla, CA) was used for statistical analysis. Significance was determined using a two-tailed unpaired Student’s t-test with a threshold of *P* < 0.05. For multivariate analysis of bladder cancer overall survival, we firstly conducted univariate analysis, and then the significant variables identified were included in the multivariate analysis.

### Data source

To analyze EIF4A3 in human pan-cancer, we obtained gene expression, clinical phenotype, and mutation data from UCSC Xena database (https://xena.ucsc.edu/) and TCGA/GTEx databases. The transcriptomic data for tumors and normal samples were processed using SangerBox (http://vip.sangerbox.com/tool.html) and converted from FPKM to TPM format through log2 conversion.

### Topology, localization and protein interaction network of EIF4A3 proteins

The human protein atlas database (http://www.proteinatlas.org) provides open-access analysis of protein expression in various subcellular levels, including tissues, single cells, immune cells, and cell lines. EIF4A3 protein subcellular localization was analyzed in A-431, U-251MG and U-2 OS cell lines and its association with endoplasmic reticulum, microtubules and nucleus was investigated. PROTTER (http://wlab.ethz.ch/protter/start/) is a tool for predicting sequence features and protein-form visualization based on experimental proteomics evidence. String (cn.string-db.org/) is an open-source database used to predict protein–protein interaction (PPI) networks with a confidence score cutoff of 0.7.

### Expression and prognostic value of EIF4A3 in pan-cancer

We utilized TIMER (https://cistrome.shinyapps.io/timer/) and UCSC Xena databases to compare EIF4A3 expression in human cancers and paired normal tissue. Additionally, we analyzed EIF4A3 expression profiles in various cancer and normal cell lines using the BioGPS database (http://biogps.org). To assess the impact of EIF4A3 expression on patient survival, we employed the Kaplan–Meier method and univariate Cox regression analysis, utilizing UCSC Xena database and SangerBox tools.

### Genes involved in RNA modification and its relationship to EIF4A3 expression

Gene expression data was downloaded from UCSC Xena database and we obtained data of 3 types of RNA modification genes (10 m1A genes, 13 m5C genes and 21m6A genes) in various human cancer types. The pearson-relationship was calculated between EIF4A3 expression and RNA modification genes. A statistical significance level of *P* < 0.05 was applied.

### Examining EIF4A3 expression’s correlation with immune and molecular subtypes

TISIDB is a database that combines various data types to study the interaction between tumors and the immunology (http://cis.hku.hk/TISIDB/index.php). It was utilized to investigate the relationship between EIF4A3 expression and the immune or molecular subtypes of various cancers. A statistical significance level of *P* < 0.05 was applied.

### Examining the EIF4A3 expression’s correlation with immune checkpoint genes

Using the UCSC Xena database, we analyzed the correlation between EIF4A3 expression and immune checkpoint genes. These genes were previously classified into inhibitory and stimulatory categories in a published study^13^.

### Examining the EIF4A3 expression’s correlation with tumor immune microenvironment in pan-cancer

The SangerBox tool was used to calculate stromal, immune, and ESTIMATE scores in human cancers. Only results with a significance level of *P* < 0.05 were analyzed. Additionally, the IOBR package’s Timer method was utilized to re-evaluate the B cell, T cell CD4, T cell CD8, Neutrophil, Macrophage, and DC invasion scores of each patient in each tumor based on EIF4A3 gene expression^14,15^.

### Examining the relationship between EIF4A3 gene alteration and tumor immunity

By utilizing the tumor immune dysfunction and exclusion database (TIDE), the effectiveness of immune checkpoint blockade treatment can be predicted (http://tide.dfci.harvard.edu/). The predictive value of EIF4A3 was compared to other previously published markers in immune checkpoint blockade cohorts within TIDE, and its relationship with responses to immune checkpoint blockade treatments and T cell dysfunction levels were evaluated in various cohorts. Additionally, the cBioportal database was used to analyze EIF4A3 copy number alterations and genetic alterations in different cancers (http://www.cbioportal.org/).

### Ethics approval and consent to participate

The acquisition of bladder cancer and adjacent tissue samples was authorized by the local committee, following patient consent. Other data and analyses were based on previous published studies; thus, no ethical approval and patient consent are required.

## Results

### EIF4A3 is upregulated in bladder cancer and associated with prognosis

**Figure 1:**
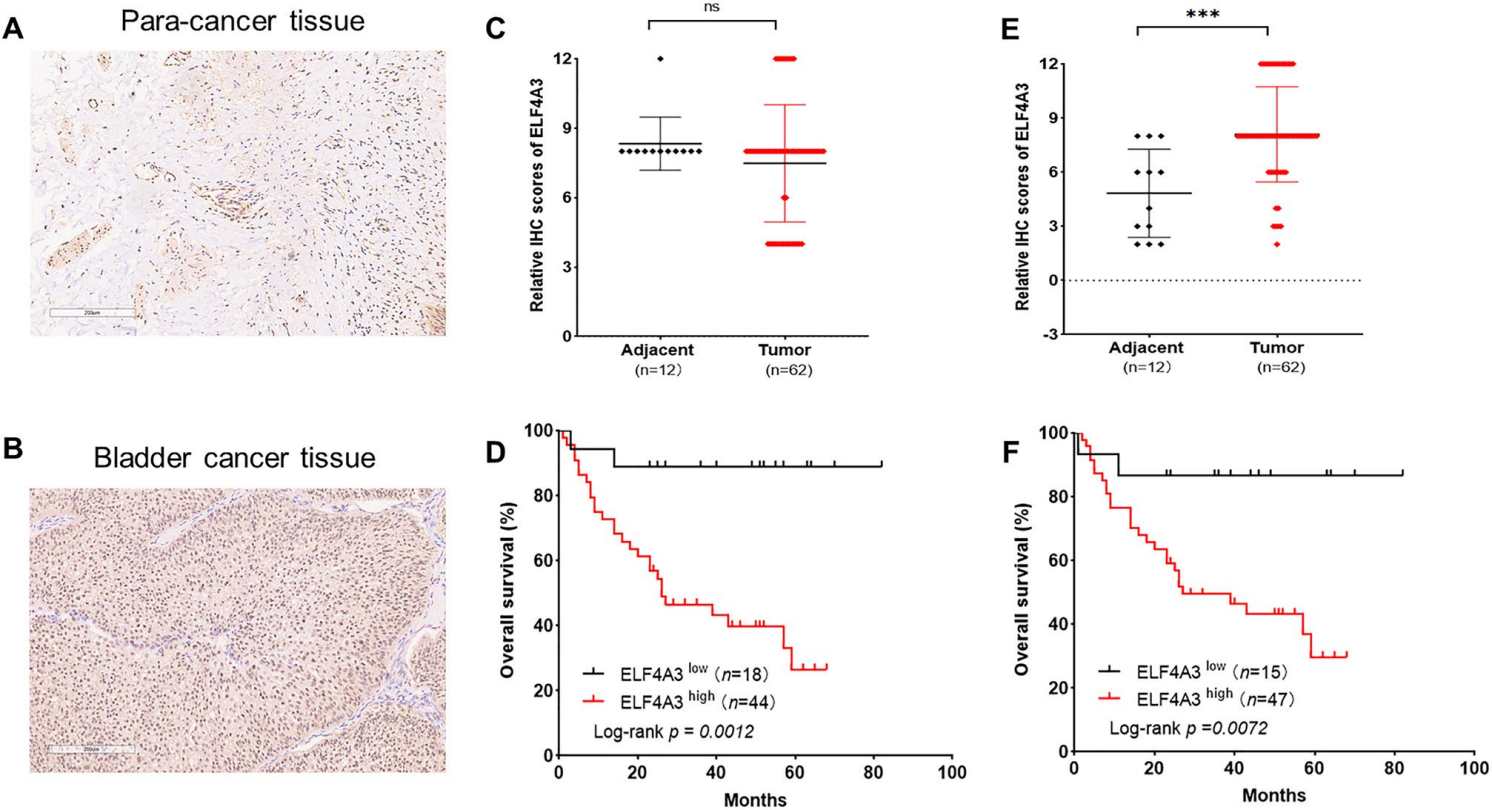
EIF4A3 is upregulated in bladder cancer and associated with prognosis. (**A**, **B**) Representative images of immunohistochemical staining showed the expression level of EIF4A3 in bladder cancer tissues and adjacent para-cancer tissues. (**C, D**) The expression level of **cytoplasmic** EIF4A3 is similar in bladder cancer tissues compared to adjacent para-tumor tissues. Kaplan–Meier survival analysis demonstrated that the upregulation of cytoplasmic EIF4A3 expression levels was significantly correlated with a poorer overall survival rate in bladder cancer. (E–F) The expression level of **nucleus** EIF4A3 was significantly higher in bladder cancer tissues compared to adjacent para-tumor tissues. Kaplan–Meier survival analysis demonstrated that the upregulation of nucleus EIF4A3 expression levels was significantly correlated with a poorer overall survival rate in bladder cancer.

To verify the expression level of EIF4A3 and its prognostic value, we collected 62 bladder cancer tissue samples and 12 adjacent para-cancer tissues along with clinical data. An immunohistochemical staining assay was conducted. Overall, the expression level of nucleus EIF4A3 was significantly higher in bladder cancer tissues compared to adjacent para-tumor tissues. Additionally, Kaplan–Meier survival analysis demonstrated that the upregulation of both nucleus and cytoplasmic EIF4A3 expression levels was significantly correlated with a poorer overall survival rate in bladder cancer (Fig. 1). Then we analysed the correlation between ELF4A3 expression and clinicopathological characteristics including patient sex, age, tumor grade, tumor size, TNM stage, CD8 expression and PDL1 expression. However, no significant association was observed between both nucleus and cytoplasmic EIF4A3 expression levels and clinicopathological characteristics (Supplementary Tables 1 and 2). Finally, we performed univariate and multivariate analyses to assess the factors associated with overall survival in patients with bladder cancer. We firstly conducted univariate analysis, and then the significant variables identified were included in the multivariate analysis.

The results revealed a significant correlation that expression of EIF4A3 in the cytoplasm (HR = 17.829, 95% CI 2.300–138.173, *P* = 0.006) and TNM stage (HR = 2.190, 95% CI 1.002–4.786, *P* = 0.049) was significantly related with the risk of death from bladder cancer (Supplementary Table 3). Additionally, we found that EIF4A3 expression in the nucleus was significantly associated with overall survival (HR = 4.848, 95% CI 1.080–21.760, *P* = 0.039, Supplementary Table 4).

### Topology, localization and protein interaction network of EIF4A3 proteins

**Figure 2:**
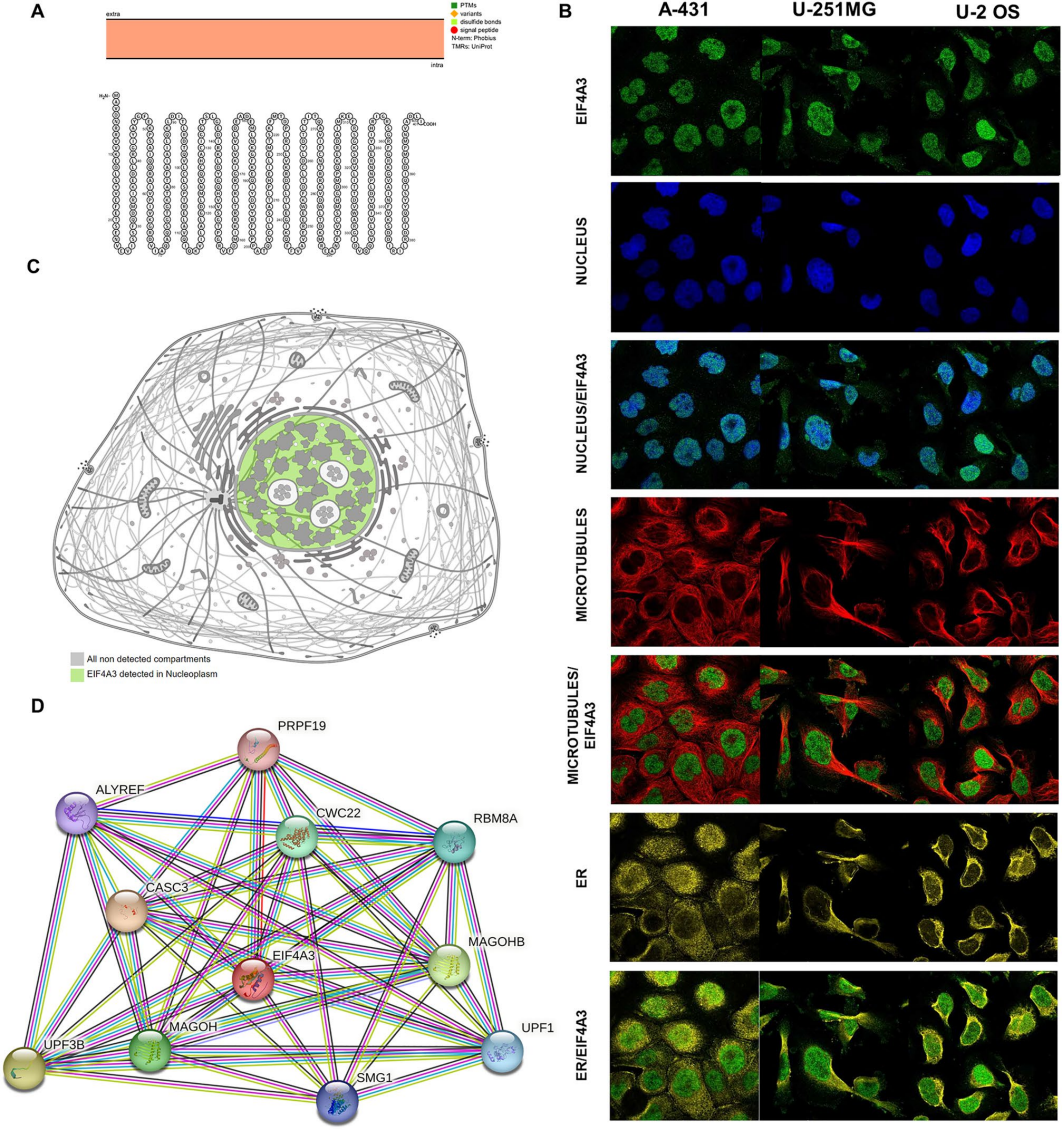
Topology, localization and protein interaction network of EIF4A3 proteins. (**A**) The topology of EIF4A3 proteins is depicted, which indicates that this protein predominantly localizes to intracellular membranes. (**B**) The intracellular localization pattern of EIF4A3 protein was determined using immunofluorescence staining, revealing nuclear, microtubular, and endoplasmic reticulum staining in HEL, REH, and U-2 OS cell lines, as extracted from the Human Protein Atlas database. (**C**) The distribution of EIF4A3 expression, which was mainly detected in the nucleoplasm and cytosol. (**D**) The protein interaction network of EIF4A3 proteins, highlighting the various protein–protein interactions and associations gleaned from the String database.

To investigate the role of EIF4A3 in human cancers, we performed a pan-cancer analysis and examined its topology, localization, and protein interaction network. The intracellular membrane localization of EIF4A3 was observed physiologically (Fig. 2A). We also analyzed the intracellular protein localization of EIF4A3 using data from The Human Protein Atlas database. The results showed that EIF4A3 was distributed heterogeneously at nucleus, microtubes, and ER in A-431, U-251MG, and U-2 OS cell lines (Fig. 2B). The EIF4A3 protein was mainly detected in nucleoplasm based on the cell sketch (Fig. 2C). Protein–protein interaction analysis revealed that EIF4A3 was associated with PRPF19, ALYREF, CWC22, RBM8A, CASC3, MAGOHB, UPF3B, MAGOH, SMG1, and UPF1 (Fig. 2D).

### Expression of EIF4A3 in pan-cancer

**Figure 3:**
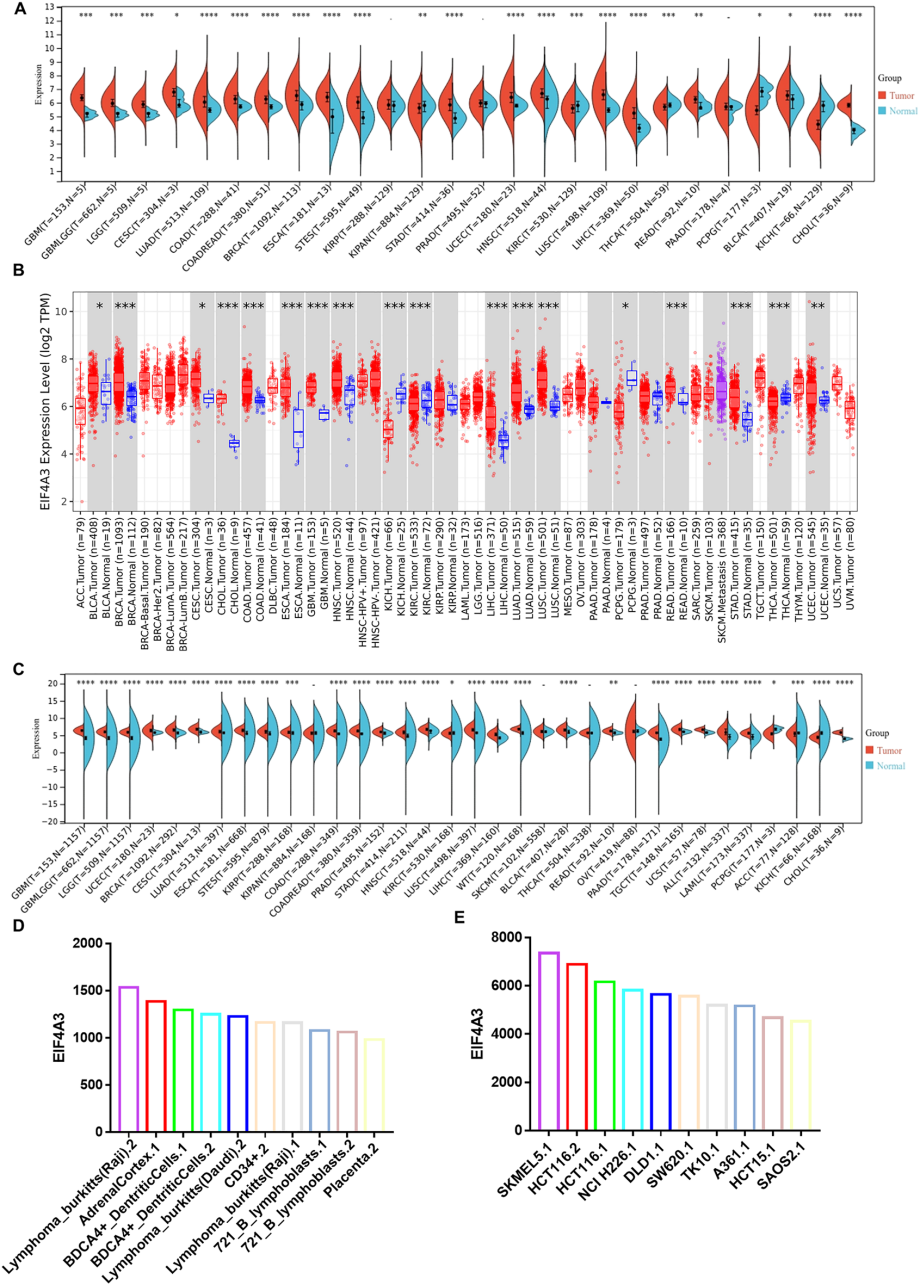
The expression profile of EIF4A3 in pan-cancer. (**A**) The expression levels of EIF4A3 in human cancers, as extracted from the cancer genome atlas (TCGA) database. (**B**) The expression levels of EIF4A3 in human cancers, as determined by the TIMER 2.0 database, which is also based on the TCGA database. (**C**) The expression levels of EIF4A3 in human cancers as well as normal tissues, based on data from both the TCGA and Genotype-Tissue Expression (GTEx) databases. (**D**) The expression levels of EIF4A3 in normal and cancer cell lines, providing insight into the tissue-specific expression patterns of this protein across multiple cancer types. *p < 0.05; **p < 0.01; ***p < 0.001.

To investigate the correlation between EIF4A3 and cancers, we analyzed its mRNA expression levels in various tissues using multiple databases. TCGA database was utilized to obtain EIF4A3 expression patterns in cancers and paraneoplastic tissues (Fig. 3A). Our findings indicate that EIF4A3 expression is significantly higher in 18 cancers, including GBM, GBMLGG, LGG, CESC, LUAD, COAD, COADREAD, BRCA, ESCA, STES, STAD, UCEC, HNSC, LUSC, LIHC, READ, **BLCA** and CHOL (*P* < 0.05, cancer type abbreviations are shown in supplementary Table 5). Conversely, EIF4A3 expression is lower in 5 cancers, such as KIPAN, KIRC, THCA, PCPG and KICH. Additionally, we verified the expression of EIF4A3 in different cancers using TIMER 2.0 database, which is based on TCGA database (Fig. 3B). Furthermore, we combined the data from TCGA and GTEx database (Fig. 3C) and found that EIF4A3 expression is upregulated in 27 cancers, including GBM, GBMLGG, LGG, UCEC, BRCA, CESC, LUAD, ESCA, STES, KIRP, COAD, COADREAD, PRAD, STAD, HNSC, KIRC, LUSC, LIHC, WT, BLCA, READ, PAAD, TGCT, UCS, ALL, LAML and CHOL. Conversely, EIF4A3 expression is significantly lower in only 3 cancers, including PCPG, ACC and KICH. Overall, our analysis of TCGA and GTEx data suggests that EIF4A3 is overexpressed in most cancer types.

We used the BioGPS database to determine the expression level of EIF4A3 in both normal tissues and cancer cell lines. Figure 3D shows the top 10 normal tissues with the highest expression level of EIF4A3, while Fig. 3E displays the top 10 cancer cell lines with the highest expression level of EIF4A3. Our findings indicate that EIF4A3 expression is generally higher in cancer cell lines compared to normal tissues.

### Prognostic value of EIF4A3 in pan-cancer

**Figure 4:**
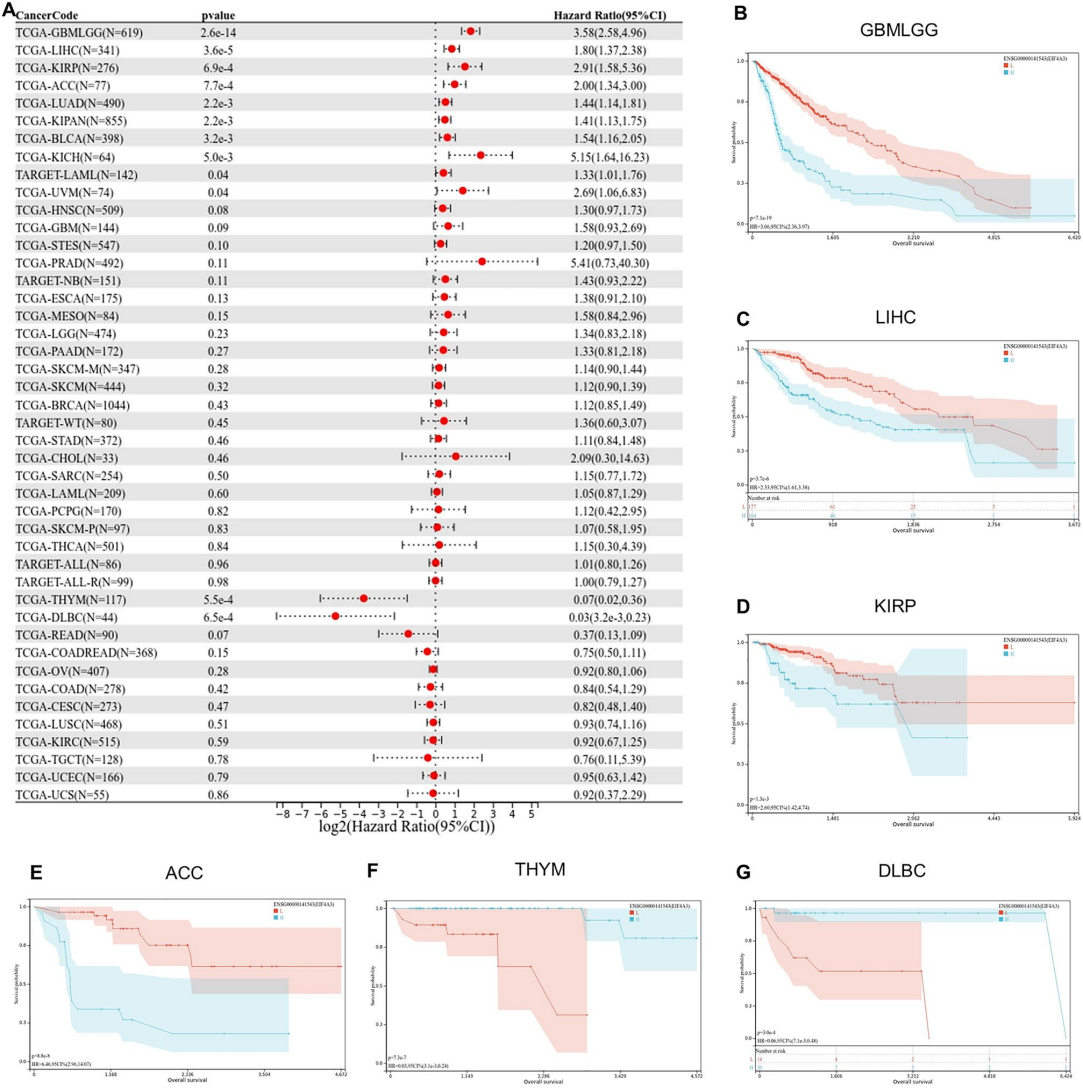
The prognostic value of EIF4A3 in pan-cancer. (**A**) The relationship between EIF4A3 expression levels and tumor overall survival across multiple cancer types. (**B**–**G**) Kaplan–Meier survival curves for various cancer types, showing the association between EIF4A3 expression levels and OS. These cancer types include GBMLGG, LGG, KIRP, KIPAN, LIHC and TCGA-MESO.

We assessed the effect of EIF4A3 expression on the clinical prognosis of patients with multiple cancers from UCSC Xena database. Cox regression model was used to analyze the Overall survival of each cancer (Fig. 4A). Higher expression of EIF4A3 was significantly associated with a poorer outcome in ten cancers including TCGA-GBMLGG, TARGET-LAML, TCGA-LUAD, TCGA-KIRP, TCGA-KIPAN, TCGA-LIHC, **TCGA-BLCA**, TCGA-UVM, TCGA-ACC and TCGA-KICH. Conversely, lower expression of EIF4A3 was significantly associated with a poorer outcome in two cancers including TCGA-THYM and TCGA-DLBC. We also conducted Kaplan–Meier analysis to verify the impact of EIF4A3 expression on patient prognosis in several cancers (Fig. 4B–G). Therefore, EIF4A3 may mainly act as a cancer-promoting gene in different cancer types.

### RNA modification related genes and its relationship to EIF4A3 expression

**Figure 5:**
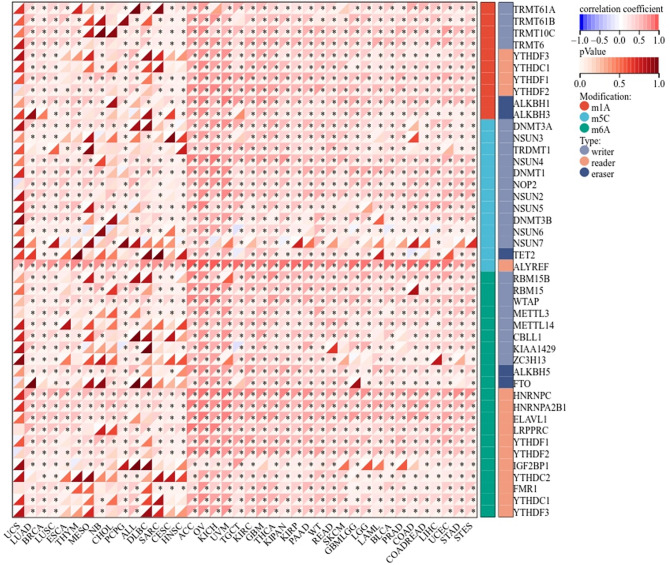
EIF4A3 demonstrated a significant correlation with the majority of RNA modification genes across several types of cancer.

We assessed the relationship between EIF4A3 expression and RNA modification genes with multiple cancers from UCSC Xena database. Expression data of three types of RNA modification genes including 10 m1A genes, 13 m5C genes and 21m6A genes were extracted and pearson’s correlation analysis were conducted. As a novel m6A suppressor, EIF4A3 was correlated with most RNA modification genes in multiple cancers (Fig. 5). Particularly in bladder cancer, EIF4A3 was correlated with all m6A related genes, suggesting its potentially comprehensive role to regulate gene fate as m6A suppressor.

### The immune and molecular subtypes related to EIF4A3 expression in pan-cancer

**Figure 6:**
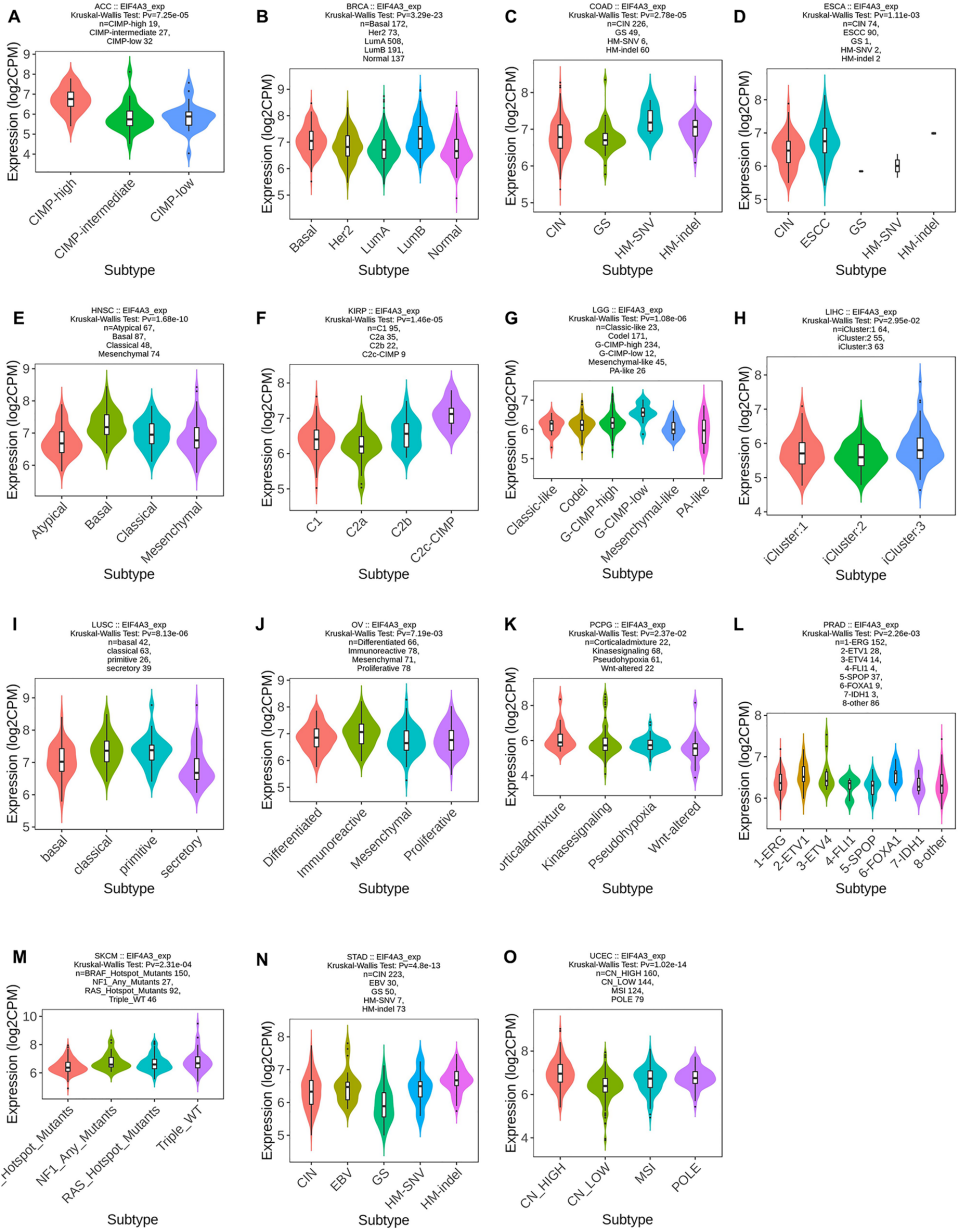
The correlation between EIF4A3 and molecular subtypes across various human cancers. (**A**) Analysis of its association in ACC; (**B**) analysis of its association in BRCA; (**C**) analysis of its association in COAD; (**D**) analysis of its association in ESCA; (**E**) analysis of its association in HNSC; (**F**) analysis of its association in KIRP; (**G**) analysis of its association in LGG; (**H**) analysis of its association in LIHC; (**I**) analysis of its association in LUSC; (**J**) analysis of its association in OV; (**K**) analysis of its association in PCPG; (**L**) analysis of its association in PRAD; (**M**) analysis of its association in SKCM; (**N**) analysis of its association in STAD; (**O**) analysis of its association in UCEC.

**Figure 7:**
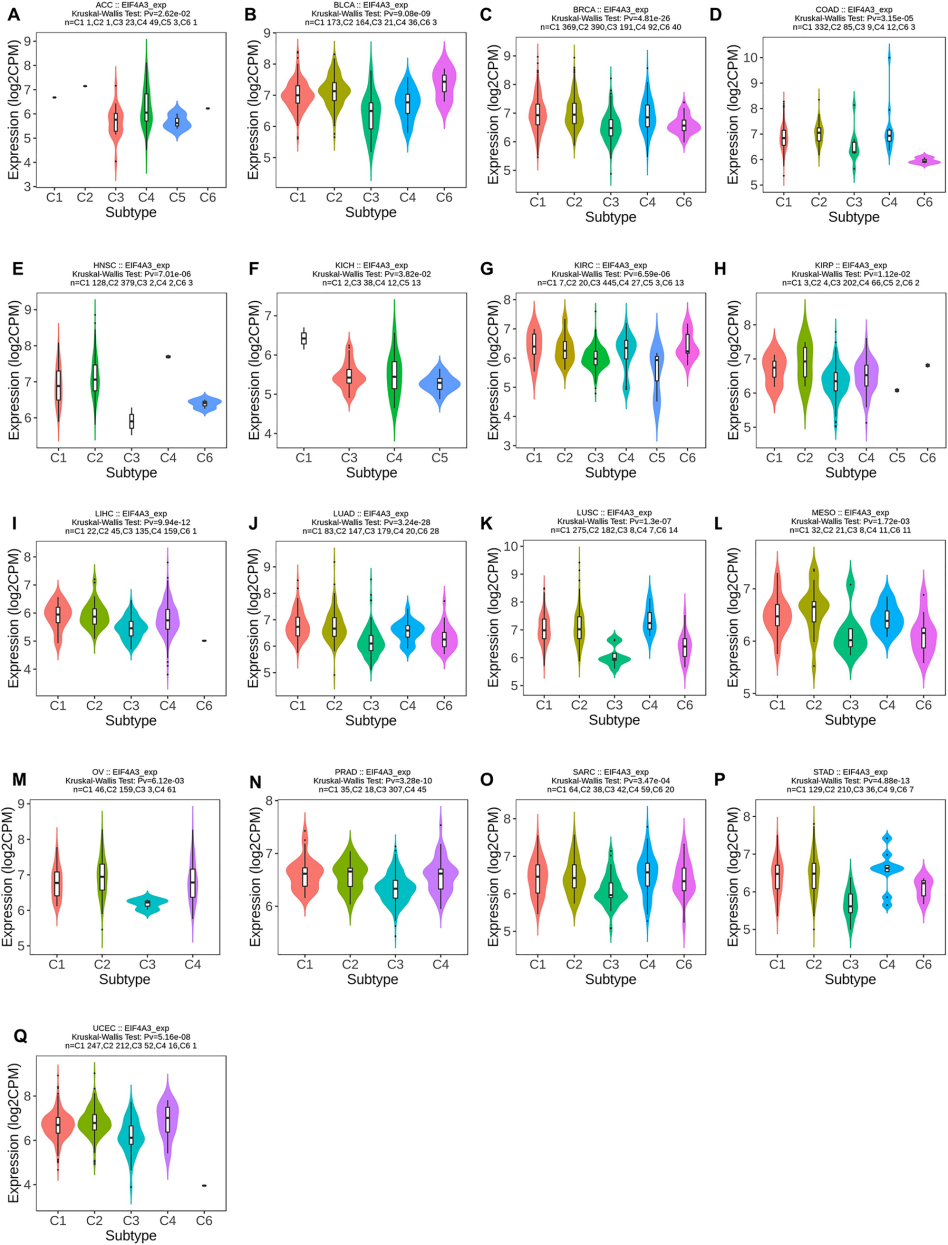
The correlation between EIF4A3 and and immune subtypes across various human cancers. (**A**) Analysis of its association in ACC; (**B**) analysis of its association in BLCA; (**C**) analysis of its association in BRCA; (**D**) analysis of its association in COAD; (**E**) analysis of its association in HNSC; (**F**) analysis of its association in KICH; (**G**) analysis of its association in KIRC; (**H**) analysis of its association in KIRP; (I) analysis of its association in LIHC; (**J**) analysis of its association in LUAD; (**K**) analysis of its association in LUSC; (**L**) analysis of its association in MESO; (**M**) analysis of its association in OV; (**N**) analysis of its association in PRAD; (**O**) analysis of its association in SARC; (**P**) analysis of its association in STAD; (**Q**) analysis of its association in UCEC.

We used TISIDB database to investigate the correlation between immune and molecular subtypes and EIF4A3 expression in various cancers^16^. We found a significant association between molecular subtype and EIF4A3 expression in ACC, BRCA, COAD, ESCA, HNSC, KIRP, LGG, LIHC, LUSC, OV, PCPG, PRAD, SKCM, STAD and UCEC (Fig. 6). Additionally, we observed a significant connection between EIF4A3 expression and the six immune subtypes (C1-wound healing, C2-IFN-gamma dominant, C3-inflammatory, C4-lymphocyte depleted, C5-immunologically quiet and C6-TGF-b dominant) in 17 types of cancer including ACC, BLCA, BRCA, COAD, HNSC, KICH, KIRC, KIRP, LIHC, LUAD, LUSC, MESO, OV, PRAD, SARC, STAD and UCEC (Fig. 7). Notably, EIF4A3 expression was significantly related to both immune and molecular subtypes in several cancer types such as ACC, BRCA, COAD, HNSC, KIRP, LIHC, LUSC, OV, PRAD, STAD and UCEC, indicating that EIF4A3 may be a crucial factor in these cancers and further research is required to confirm its potential as a precise treatment target.

### Immune check point genes related to EIF4A3 expression in pan-cancer

**Figure 8:**
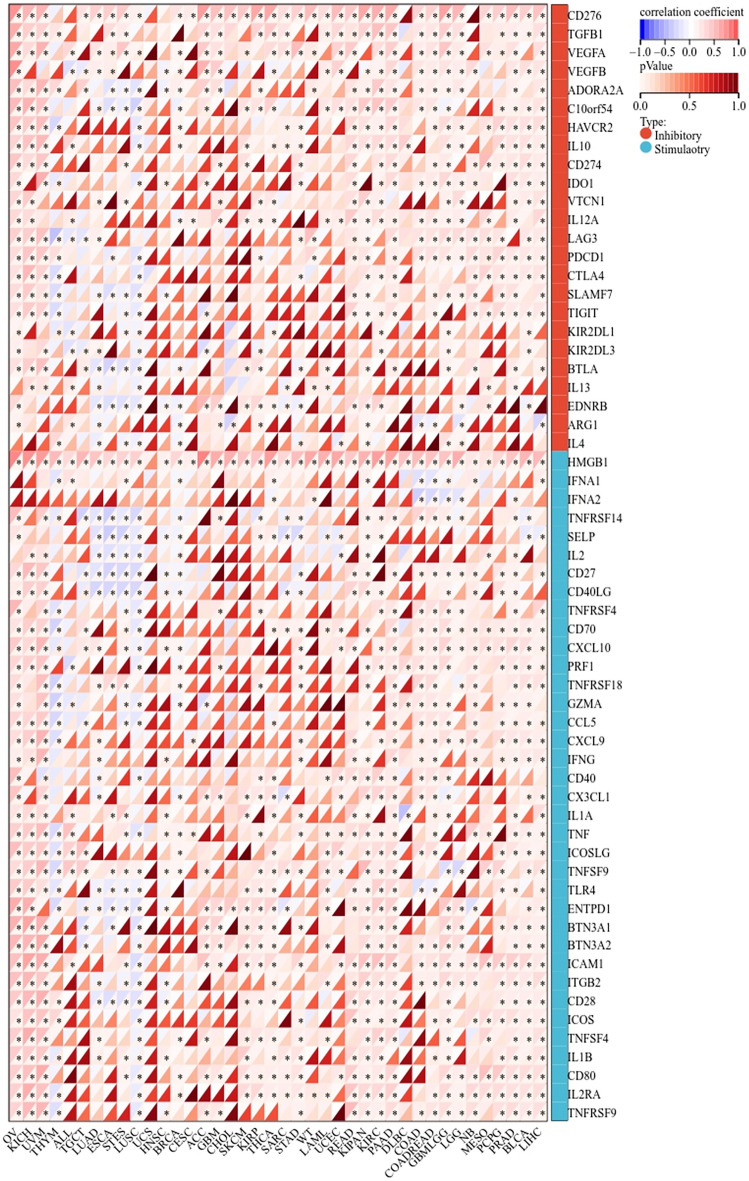
The potential correlation between EIF4A3 and immune checkpoint genes, comprising of 24 inhibitory genes and 36 stimulatory genes, across forty human cancers *p < 0.05; **p < 0.01; ***p < 0.001.

We investigated the potential of EIF4A3 in immunotherapy by studying its relationship with immune checkpoint genes in human cancers. Data from UCSC Xena database was used to explore the expression of EIF4A3 and 24 inhibitory genes and 36 stimulatory genes in 40 human cancers. The correlation between EIF4A3 and immune checkpoint genes was calculated and displayed in Fig. 8. A significant relationship was found between EIF4A3 and most immune checkpoint genes in several cancers such as OV, KICH, UVM, LIHC, BLCA, etc. In BLCA, 43 out of 60 immune checkpoint genes showed significant correlation with EIF4A3 expression. This suggests that EIF4A3 may regulate the expression of these immune checkpoint genes in different pathways, and thus have potential effect on immunotherapy. Our analysis indicates that EIF4A3 may act as a potential biomarker for predicting the outcome of immunotherapy or as a new target to improve the effect of immunotherapy.

### Tumor immune microenvironment and EIF4A3 in pan-cancer

**Figure 9:**
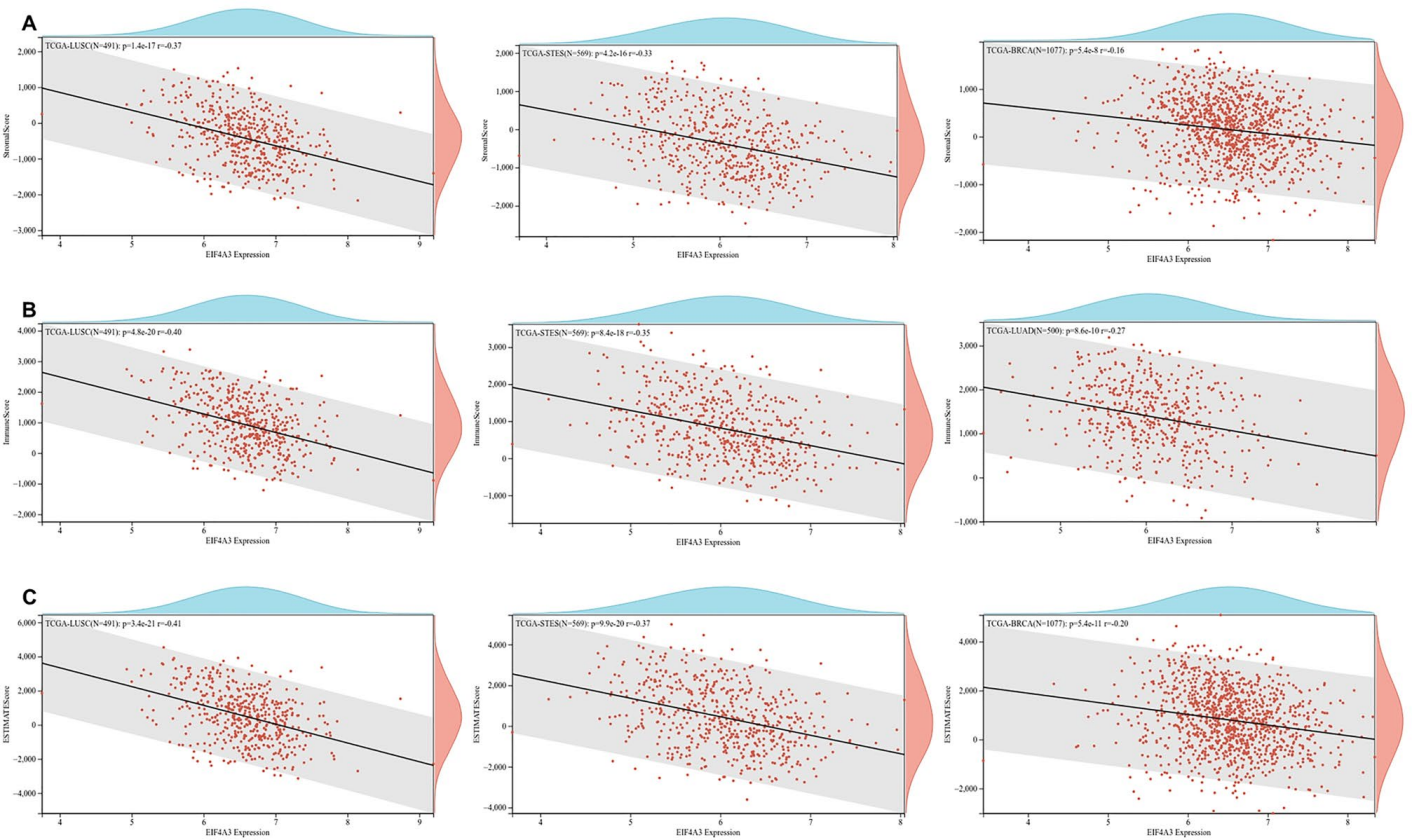
The potential associations between EIF4A3 expression and immune infiltration scores across numerous human cancers, (**A**) the top 3 cancer types with the most significant relationship between EIF4A3 expression and immune score was presented; (**B**) the top 3 cancer types with the most significant relationship between EIF4A3 expression and stromal score was presented; (**C**) the top 3 cancer types with the most significant relationship between EIF4A3 expression and ESTIMATE score was presented.

**Figure 10:**
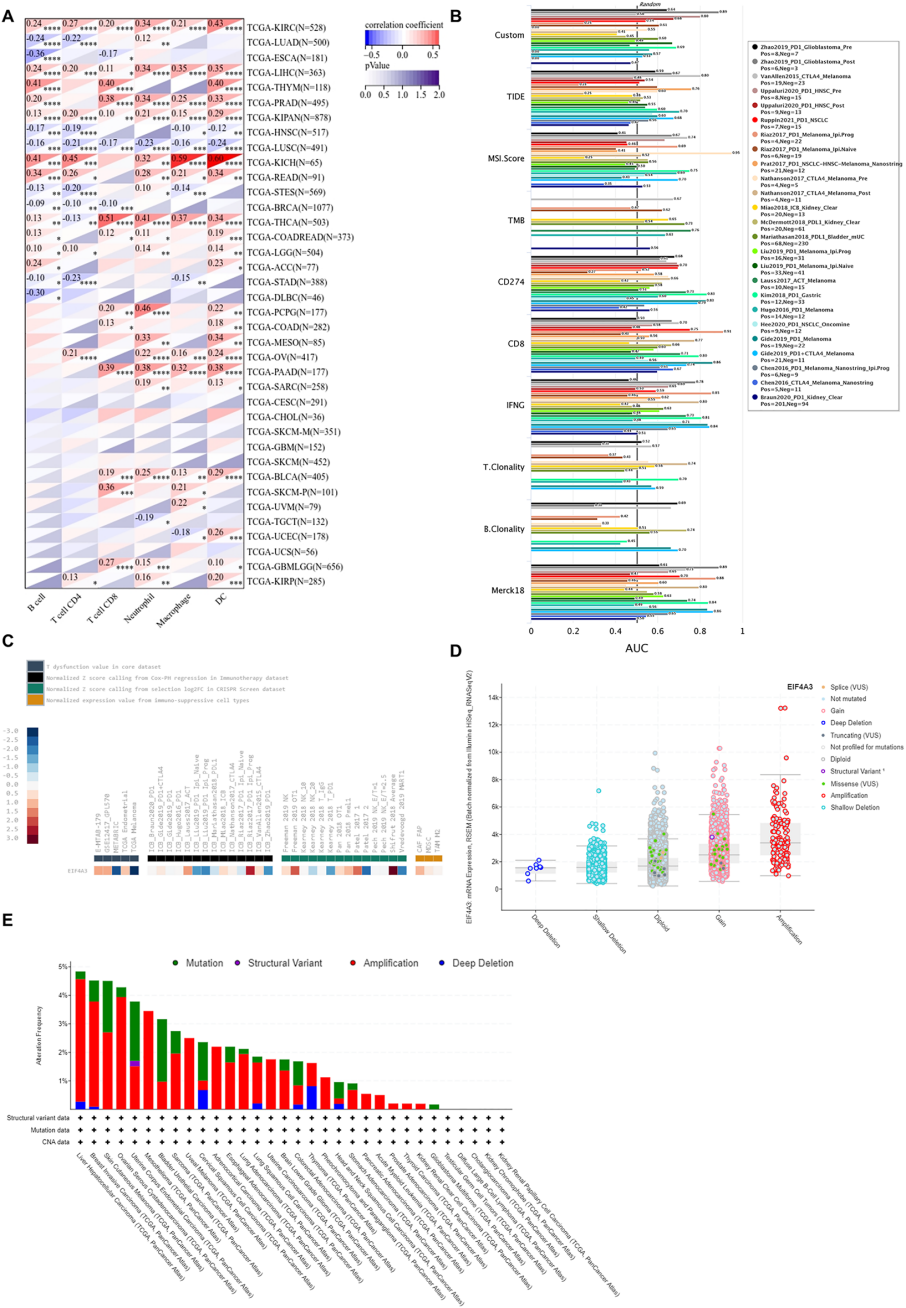
The potential correlation between EIF4A3 gene alteration and tumor immunity. (**A**) The potential association between infiltration of immune cells and EIF4A3 expression. (**B**) The effectiveness of EIF4A3 as a biomarker compared to traditional immunotherapy biomarkers. (**C**) The relationship between EIF4A3 expression with T cell dysfunction, immunotherapy outcome, natural killer cell anti-tumor activity in CRISPR screen dataset and expression value from immune-suppressive cell types. (**D**) The copy-number alterations of EIF4A3, where amplification was the most common type of copy-number alteration in EIF4A3. (**E**) The genomic alteration of EIF4A3, where amplification was the most frequent alteration type.

Recent evidence suggests that the tumor immune microenvironment plays a crucial role in the development and progression of human cancers. To investigate the relationship between EIF4A3 expression and immune signatures, we calculated the stromal score, immune score, and ESTIMATE score. Our analysis of 44 types of cancer revealed that LUSC, STES, and LUAD had the strongest association between EIF4A3 expression and immune score (Fig. 9A). Similarly, LUSC, STES, and BRCA had the strongest association between EIF4A3 expression and stromal score (Fig. 9B). Surprisingly, the top three tumors with the most significant association between EIF4A3 expression and ESTIMATE score were the same as stromal score, including LUSC, STES, and BRCA (Fig. 9C). These findings suggest that EIF4A3 expression levels in LUSC, STES, LUAD and BRCA were positively correlated with the infiltration of immune cells and stromal cells. Additionally, we analyzed the potential relationship between EIF4A3 expression and infiltration of several important types of immune cells (Fig. 10A). Our analysis of 38 types of human cancer revealed that EIF4A3 expression had a significant relationship with cell infiltration in 31 types of human cancer, with a strong correlation with B cell in 19 cancer types, CD4 T cell in 15 cancer types, CD8 T cell in 16 cancer types, neutrophil in 19 cancer types, macrophage in 17 cancer types, and DC in 23 cancer types.

### EIF4A3 gene alteration and relationship with tumor immunity

We evaluated EIF4A3 as a potential biomarker for immunotherapy by comparing it with traditional biomarkers in TIDE dataset. Our findings showed that EIF4A3 had an AUC greater than 0.5 in 13 out of 25 immunotherapy cohorts (Fig. 10B), indicating its potential as an immunotherapy biomarker. Interestingly, EIF4A3 demonstrated similar predictive ability to MSI.Score and may even outperform TMB, T. Clonality, and B. Clonality.

We evaluated the correlation between EIF4A3 expression and immunotherapy response in multiple cohorts (Fig. 10C). High EIF4A3 expression was associated with poor outcomes of anti-PD-1 treatment in melanoma and glioblastoma, but showed positive outcomes of adoptive T cell therapy and anti-PD-1 treatment in melanoma. Notably, two studies received totally opposite outcome while predicting anti-PD-1 treatment outcome according to EIF4A3 expression in melanoma^17,18^. This discrepancy may be due to study heterogeneity, as Liu’s study included 144 melanoma patients (60 with prior exposure to ipilimumab) treated with nivolumab or pembrolizumab, while Rias’s study included 68 melanoma patients (35 with prior progression on ipilimumab) treated with nivolumab. The TIDE dataset showed a strong negative correlation between T cell dysfunction and EIF4A3 expression in breast cancer and melanoma. Additionally, microarray analysis detected high EIF4A3 expression in FAP (+) tumor-associated fibroblasts.

We next assessed the genomic changes in EIF4A3 using cBioPortal database. Amplification was the most prevalent type of gene alteration, followed by mutation and deep deletion (Fig. 10E). We also analyzed the copy-number alterations of EIF4A3 (Fig. 10D), with amplification being the most frequent type, followed by gene gain, diploid, shallow deletion, and deep deletion.

## Discussion

A growing body of evidence indicates a significant association between EIF4A3 and the progression of a broad spectrum of malignancies. The research conducted by Huang et al. has revealed that EIF4A3 promotes overexpression of circZFAND6 by specifically binding to the upstream regions of its pre-mRNA in breast cancer and additionally, the suppression of EIF4A3 expression has been shown to impede tumor proliferation and metastasis^19^. In liver cancer, EIF4A3 has been found to induce dysfunction of CD8+T cells by promoting the formation of circCCAR1, leading to the development of resistance mechanisms against PD-1 inhibitors in tumor cells^20^. Recently a significant achievement has been made in the field of epigenetics that m6A specificity is globally regulated by “suppressors” that prevent m6A deposition in unmethylated regions of the transcriptome and exon junction complexes, with EIF4A3 as the core factor, have been identified as m6A suppressors that protect exon junction-proximal RNA within coding sequences from methylation and regulate mRNA stability through m6A suppression^7^. This discovery reveals the important role of EIF4A3 as a m6A regulator which could extensively modulate gene expression outcome, providing new hope for gene therapies. To explore the prognostic and immune significance of EIF4A3 in human cancers, a pan-cancer analysis was urgently needed, and our findings were further validated in bladder cancer tissues.

We initially investigated the expression and prognostic impact of EIF4A3 in bladder cancer. Our results confirmed that the upregulation of EIF4A3 occurs in bladder cancer, and that patients with increased expression levels of EIF4A3 exhibit a poorer prognosis. We were thus prompted to consider that EIF4A3 might function as an oncogene in human cancer. In order to verify its role in human cancers, we conducted a pan-cancer analysis. Of the 34 tumors we examined in GTEx and TCGA databases, EIF4A3 gene expression level was significantly higher than normal tissues in 27 kinds of tumors and lower in only 3 kinds of tumors. Simultaneously, our findings on the relationship between EIF4A3 and tumors were supported by several previous studies. Zheng et al. reported that EIF4A3 facilitates the carcinogenesis and development of triple-negative breast cancer by promoting biogenesis of circSEPT9^21^. Moreover, a study in recent years unveiled the oncogenic function of EIF4A3 in epithelial ovarian cancer^22^. Apart from the oncogenic role of EIF4A3 in hunman cancers, it is also reported to modulate neurodevelopmental disorders and embryonic stem cell identity^23,24^. The comprehensive regulation role of EIF4A3 may be caused by its wide range of effects on gene fate such as m6A suppression, mRNA splicing and RNA-binding protein, implying that spatiotemporal specificity of EIF4A3 RNA may play a critical role in determining and influencing diverse cell fates in disease progression. In our analysis, we also found a significant association between EIF4A3 and nearly all m6A regulators in the majority of human cancers studied. These findings suggested that EIF4A3 may be a potential biomarker to predict the prognosis of different malignancies while more studies are necessary to further determine the exact role of EIF4A3 in human cancers. While our study offers valuable insights into the role of EIF4A3 in human cancers, another study endeavors to provide a comprehensive perspective on kinase expression patterns across a diverse spectrum of cancer types, and both sets of findings possess substantial scientific merit^25^.

To investigate the putative contribution of EIF4A3 to tumor progression and immune response, we probed the expression of EIF4A3 in diverse molecular and immune subtypes in human cancers. Our findings indicate significant difference in EIF4A3 expression levels across several molecular and immune subtypes in most cancer types, suggesting that EIF4A3 could represent a reliable pan-cancer diagnostic biomarker while also playing a crucial role in the regulation of the tumor immune microenvironment. To further elucidate the relationship between EIF4A3 and the tumor immune microenvironment, we calculated immune infiltration scores and explored immune cell infiltration. Our analysis revealed a significant correlation between EIF4A3 expression levels and immune scores in 23 out of 44 cancer types, indicating that EIF4A3 is closely associated with the tumor immune microenvironment in human cancers. We then conducted an in-depth analysis of immune cell infiltration in the tumor microenvironment, revealing a strong correlation between EIF4A3 expression levels and B cell, neutrophil and DC infiltration in most cancer types. Tumor-infiltrating B cells have been shown to promote antitumor immunity by aiding in the maturation of tumor-associated tertiary lymphoid structures, which have been identified as important predictors of immunotherapy response^26^. Given the diverse nature of the relationship between immune cells and EIF4A3 expression in human cancers, we hypothesize that there is a complex correlation between the antitumor or pro-tumor response of immune cells and EIF4A3 expression. In our analysis of the intricate relationship between bladder cancer and immune cells, a remarkable augmentation of CD8-positive T cells was observed in bladder cancer patients (Fig. 10A). Building upon the seminal work conducted by Chen et al., an influential association was unveiled between the infiltration of CD8-positive T cells in bladder cancer tissue and the prognostic outlook for patients^27,28^. These findings harmonize seamlessly with our own results, thereby extending the horizons and emphasizing the paramount significance of CD8-positive T cells in the comprehensive prognostic assessment of bladder cancer. Further studies are necessary to illuminate the underlying mechanisms. Meanwhile, our analysis of the correlation between EIF4A3 and immune checkpoint genes indicates that EIF4A3 may play a pivotal role in the regulation of tumor immunity within complex molecular pathways. Moreover, our study suggests that EIF4A3 has potential as a superior immunotherapy biomarker compared to traditional biomarkers such as TMB, T. Clonality, and B. Clonality in approximately half of the immunotherapy cohorts examined. A strong negative correlation between T cell dysfunction and EIF4A3 expression was observed in breast cancer and melanoma, suggesting the potential of EIF4A3 as an immune regulator. We also detected higher expression levels of EIF4A3 in FAP (+) tumor-associated fibroblasts, indicating a potential link between the tumor microenvironment and EIF4A3. In conclusion, our findings reveal that EIF4A3 is strongly correlated with tumor immunity across varying types of human cancers and may serve as a novel predictor of immunotherapy response. Further studies are necessary to elucidate the underlying molecular mechanisms of EIF4A3 in tumor progression and immunity.

We also performed a gene alteration analysis of EIF4A3, revealing that amplification was the most frequent alteration type of EIF4A3, followed by mutation and deep deletion. In addition to gene alteration analysis, we also analyzed copy-number alterations of EIF4A3. Amplification was found to be the most common type of copy-number alteration, followed by gene gain, diploid, shallow deletion, and deep deletion in varying degrees.

Despite our comprehensive analysis of the prognostic and immunological roles of EIF4A3 in pan-cancer, we acknowledge that there are still some limitations to our study. Firstly, we only analyzed open-source data and could not include all data published, resulting in the possibility of publication bias and selection bias. Secondly, there is heterogeneity between various databases, and the results need further validation and confirmation. Third, it is important to note that we were unable to confirm the role of EIF4A3 in bladder cancer through experiments, as this has recently been investigated by Hu et al.^12^ They found that EIF4A3 may facilitate bladder cancer progression by promoting cell proliferation and inhibiting apoptosis and conducted a bioimformatic analysis in bladder cancer. In contrast to previous research, our study utilized bladder cancer and para-cancer tissues with clinical data analysis to validate the prognostic role of EIF4A3 in bladder cancer. Furthermore, we performed a pan-cancer analysis to unveil the role of EIF4A3 in determining and influencing diverse cell fates in human pan-cancer progression.

## Conclusion

We confirmed that the expression level of EIF4A3 is significantly higher in bladder cancer than para-cancer tissaue. Further experiments validated that patients with increased expression levels of EIF4A3 exhibit a poorer prognosis. Based on our pan-cancer analysis of EIF4A3, we identified differential expression between tumor and normal tissues, and a significant correlation between EIF4A3 expression and prognosis. Therefore, we conclude that EIF4A3 may serve as a powerful biomarker for tumor screening, prognosis, and individualized gene strategies in a broad range of malignancies. Furthermore, our analysis also revealed that EIF4A3 expression is notably associated with the tumor microenvironment and immune cell infiltration in different cancer types. Additionally, different tumor types have varying levels of response to EIF4A3’s effects on tumor immunity. As such, by elucidating the precise role of EIF4A3 in tumor development, it may enable more personalized and precise immunotherapy in the future.

### Supplementary Information

Supplementary Tables.

## Data Availability

Some database that support the findings of this study are openly available described in ‘Materials and methods’, including UCSC Xena database^29^, UALCAN^30^, TCGA^31^, GTEx^32^, human protein atlas (www.proteinatlas.org), PROTTER^33^, String^34^, TIMER^35^, BioGPS^36^, TISIDB^16^, TIDE^37^ and cBioportal database^38,39^. Other data are available from corresponding author.
