# Supplementary material for: Identification of m6A suppressor EIF4A3 as a novel cancer prognostic and immunotherapy biomarker through bladder cancer clinical data validation and pan-cancer analysis
Source: Sci Rep. 2023 Sep 30;13:16457. doi: 10.1038/s41598-023-43500-4 (PMC10542776; doi:10.1038/s41598-023-43500-4)
Supplement: Supplementary file 1 — Supplementary Tables. [file 41598_2023_43500_MOESM1_ESM.docx]

Supplementary Table 1. Correlation between cytoplasmic ELF4A3 expression and clinicopathological characteristics

|  | variables | ELF4A3 expression | | total | χ2 | p value |
| --- | --- | --- | --- | --- | --- | --- |
|  |  | low | high |  |  |  |
| SEX |  |  |  |  | 0.206 | 0.650 |
|  | Female | 4 | 6 | 10 |  |  |
|  | Man | 14 | 38 | 52 |  |  |
| Age (year) |  |  |  |  | 0.000 | 1.000 |
|  | <60 | 4 | 10 | 14 |  |  |
|  | ＞=60 | 13 | 34 | 47 |  |  |
| Age (year) |  |  |  |  | 0.008 | 0.929 |
|  | <65 | 6 | 15 | 21 |  |  |
|  | ＞=65 | 11 | 29 | 40 |  |  |
| Age (year) |  |  |  |  | 0.042 | 0.837 |
|  | <70 | 8 | 22 | 30 |  |  |
|  | ＞=70 | 9 | 22 | 31 |  |  |
| Grade |  |  |  |  | 0.000 | 1.000 |
|  | I | 2 | 5 | 7 |  |  |
|  | III | 14 | 38 | 52 |  |  |
| Tumor size |  |  |  |  | 3.686 | 0.055 |
|  | < 5cm | 6 | 28 | 34 |  |  |
|  | > = 5cm | 9 | 13 | 22 |  |  |
|  |  |  |  |  | 1.106 | 0.575 |
| T stage | T1 | 3 | 13 | 16 |  |  |
|  | T2 | 4 | 10 | 14 |  |  |
|  | T3-T4 | 9 | 18 | 27 |  |  |
| N stage |  |  |  |  | 0.321 | 0.571 |
|  | N0 | 13 | 31 | 44 |  |  |
|  | N1 | 1 | 7 | 8 |  |  |
| TNM stage |  |  |  |  | 1.744 | 0.627 |
|  | I | 2 | 4 | 6 |  |  |
|  | II | 3 | 8 | 11 |  |  |
|  | III | 8 | 12 | 20 |  |  |
|  | IV | 2 | 9 | 11 |  |  |
| CD8 |  |  |  |  | 0.309 | 0.578 |
|  | Low | 6 | 18 | 24 |  |  |
|  | High | 12 | 26 | 38 |  |  |
| PDL-1 |  |  |  |  | 0.633 | 0.426 |
|  | Low | 7 | 22 | 29 |  |  |
|  | High | 11 | 22 | 33 |  |  |
|  |  |  |  |  |  |  |

Supplementary Table 2. Correlation between nucleus ELF4A3 expression and clinicopathological characteristics

|  | variables | ELF4A3 expression | | total | χ2 | p value |
| --- | --- | --- | --- | --- | --- | --- |
|  |  | low | high |  |  |  |
| SEX |  |  |  |  | 0.000 | 1.000 |
|  | Female | 2 | 8 | 10 |  |  |
|  | Man | 13 | 39 | 52 |  |  |
| Age (year) |  |  |  |  | 0.002 | 0.968 |
|  | <60 | 4 | 10 | 14 |  |  |
|  | ＞=60 | 11 | 36 | 47 |  |  |
| Age (year) |  |  |  |  | 0.274 | 0.601 |
|  | <65 | 6 | 15 | 21 |  |  |
|  | ＞=65 | 9 | 31 | 40 |  |  |
| Age (year) |  |  |  |  | 0.932 | 0.334 |
|  | <70 | 9 | 21 | 30 |  |  |
|  | ＞=70 | 6 | 25 | 31 |  |  |
| Grade |  |  |  |  | 0.000 | 1.000 |
|  | I | 2 | 5 | 7 |  |  |
|  | III | 13 | 39 | 52 |  |  |
| Tumor size |  |  |  |  | 0.898 | 0.343 |
|  | < 5cm | 7 | 27 | 34 |  |  |
|  | > = 5cm | 7 | 15 | 22 |  |  |
|  |  |  |  |  | 0.589 | 0.745 |
| T stage | T1 | 4 | 12 | 16 |  |  |
|  | T2 | 4 | 10 | 14 |  |  |
|  | T3-T4 | 5 | 22 | 27 |  |  |
| N stage |  |  |  |  | 0.000 | 1.000 |
|  | N0 | 12 | 32 | 44 |  |  |
|  | N1 | 2 | 6 | 8 |  |  |
| TNM stage |  |  |  |  | 1.399 | 0.706 |
|  | I | 1 | 5 | 6 |  |  |
|  | II | 4 | 7 | 11 |  |  |
|  | III | 4 | 16 | 20 |  |  |
|  | IV | 2 | 9 | 11 |  |  |
| CD8 |  |  |  |  | 0.241 | 0.623 |
|  | Low | 5 | 19 | 24 |  |  |
|  | High | 10 | 28 | 38 |  |  |
| PDL-1 |  |  |  |  | 0.365 | 0.546 |
|  | Low | 6 | 23 | 29 |  |  |
|  | High | 9 | 24 | 33 |  |  |
|  |  |  |  |  |  |  |

Supplementary Table 3. Univariate and multivariate analyses of the factors correlated with overall survival of bladder carcinoma patients

| Variables | Univariate analysis | | |  | Multivariate analysis | | |  |
| --- | --- | --- | --- | --- | --- | --- | --- | --- |
|  | HR | 95%CI | p value |  | HR | 95%CI | p value |  |
| EIF4A3 Expression in Cytoplasm | 7.479 | 1.772-31.559 | 0.006 |  | 17.829 | 2.300-138.173 | 0.006 |  |
| SEX | 0.652 | 0.264-1.610 | 0.354 |  |  |  |  |  |
| Age(>=70) | 1.276 | 0.609-2.675 | 0.518 |  |  |  |  |  |
| Grade | 1.355 | 0.659-2.786 | 0.409 |  |  |  |  |  |
| Tumor size | 0.459 | 0.197-1.067 | 0.070 |  |  |  |  |  |
| T stage | 1.486 | 0.933-2.366 | 0.095 |  |  |  |  |  |
| N stage | 2.807 | 1.012-7.783 | 0.047 |  | 0.579 | 0.127-2.636 | 0.480 |  |
| TNM stage | 1.648 | 1.036-2.621 | 0.035 |  | 2.190 | 1.002-4.786 | 0.049 |  |
| CD8 | 0.806 | 0.384-1.690 | 0.568 |  |  |  |  |  |
| PDL-1 | 1.198 | 0.584-2.459 | 0.622 |  |  |  |  |  |

Supplementary Table 4. Univariate and multivariate analyses of the factors correlated with overall survival of bladder carcinoma patients

| Variables | Univariate analysis | | |  | Multivariate analysis | | |  |
| --- | --- | --- | --- | --- | --- | --- | --- | --- |
|  | HR | 95%CI | p value |  | HR | 95%CI | p value |  |
| EIF4A3 Expression in Nucleus | 5.700 | 1.349-24.085 | 0.018 |  | 4.848 | 1.080-21.760 | 0.039 |  |
| SEX | 0.652 | 0.264-1.610 | 0.354 |  |  |  |  |  |
| Age(>=70) | 1.276 | 0.609-2.675 | 0.518 |  |  |  |  |  |
| Grade | 1.355 | 0.659-2.786 | 0.409 |  |  |  |  |  |
| Tumor size | 0.459 | 0.197-1.067 | 0.070 |  |  |  |  |  |
| T stage | 1.486 | 0.933-2.366 | 0.095 |  |  |  |  |  |
| N stage | 2.807 | 1.012-7.783 | 0.047 |  | 0.908 | 0.210-3.923 | 0.897 |  |
| TNM stage | 1.648 | 1.036-2.621 | 0.035 |  | 1.724 | 0.845-3.516 | 0.134 |  |
| CD8 | 0.806 | 0.384-1.690 | 0.568 |  |  |  |  |  |
| PDL-1 | 1.198 | 0.584-2.459 | 0.622 |  |  |  |  |  |

Supplementary Table 5. Cancer type abbreviations

| TCGA-ACC | Adrenocortical carcinoma |
| --- | --- |
| TCGA-BLCA | Bladder Urothelial Carcinoma |
| TCGA-BRCA | Breast invasive carcinoma |
| TCGA-CESC | Cervical squamous cell carcinoma and endocervical adenocarcinoma |
| TCGA-CHOL | Cholangiocarcinoma |
| TCGA-COAD | Colon adenocarcinoma |
| TCGA-COADREAD | Colon adenocarcinoma/Rectum adenocarcinoma |
| TCGA-DLBC | Lymphoid Neoplasm Diffuse Large B-cell Lymphoma |
| TCGA-ESCA | Esophageal carcinoma |
| TCGA-FPPP | FFPE Pilot Phase II |
| TCGA-GBM | Glioblastoma multiforme |
| TCGA-GBMLGG | Glioma |
| TCGA-HNSC | Head and Neck squamous cell carcinoma |
| TCGA-KICH | Kidney Chromophobe |
| TCGA-KIPAN | Pan-kidney cohort (KICH+KIRC+KIRP) |
| TCGA-KIRC | Kidney renal clear cell carcinoma |
| TCGA-KIRP | Kidney renal papillary cell carcinoma |
| TCGA-LAML | Acute Myeloid Leukemia |
| TCGA-LGG | Brain Lower Grade Glioma |
| TCGA-LIHC | Liver hepatocellular carcinoma |
| TCGA-LUAD | Lung adenocarcinoma |
| TCGA-LUSC | Lung squamous cell carcinoma |
| TCGA-MESO | Mesothelioma |
| TCGA-OV | Ovarian serous cystadenocarcinoma |
| TCGA-PAAD | Pancreatic adenocarcinoma |
| TCGA-PCPG | Pheochromocytoma and Paraganglioma |
| TCGA-PRAD | Prostate adenocarcinoma |
| TCGA-READ | Rectum adenocarcinoma |
| TCGA-SARC | Sarcoma |
| TCGA-STAD | Stomach adenocarcinoma |
| TCGA-SKCM | Skin Cutaneous Melanoma |
| TCGA-STES | Stomach and Esophageal carcinoma |
| TCGA-TGCT | Testicular Germ Cell Tumors |
| TCGA-THCA | Thyroid carcinoma |
| TCGA-THYM | Thymoma |
| TCGA-UCEC | Uterine Corpus Endometrial Carcinoma |
| TCGA-UCS | Uterine Carcinosarcoma |
| TCGA-UVM | Uveal Melanoma |
| TARGET-OS | Osteosarcoma |
| TARGET-ALL | Acute Lymphoblastic Leukemia |
| TARGET-NB | Neuroblastoma |
| TARGET-WT | High-Risk Wilms Tumor |
